# Strangulated internal hernia caused by an iatrogenic peritoneal band after total laparoscopic hysterectomy—A caveat to consider retroperitoneum closure

**DOI:** 10.1002/ccr3.6550

**Published:** 2022-11-06

**Authors:** Shizuka Sakurai, Yukio Suzuki, Koichi Nagai, Yumi Ishidera, Kazuya Nakagawa, Etsuko Miyagi

**Affiliations:** ^1^ Department of Obstetrics and Gynecology Yokohama City University Graduate School of Medicine Yokohama Japan; ^2^ Division of Gynecologic Oncology, Department of Obstetrics and Gynecology Columbia University Vagelos College of Physicians and Surgeons New York New York USA; ^3^ Department of Gastroenterological Surgery Yokohama City University Graduate School of Medicine Yokohama Japan

**Keywords:** complication, internal hernia, laparoscopic hysterectomy, retroperitoneum closure

## Abstract

Suturing the retroperitoneum is a common technique in laparoscopic hysterectomy, which is reported to reduce vaginal cuff infection and organ evisceration in case vaginal cuff dehiscence occurs. However, physicians should take into account that it may cause internal hernia.

## 1 INTRODUCTION

Intestinal obstruction is a considerably rare complication of gynecologic laparoscopy, with an incidence of 0.036%.^1^ Regarding laparoscopic hysterectomy for benign disease, the incidence of postoperative intestinal obstruction is reported to be 0.4%.^2^ The incidence rate of internal hernia after gynecologic laparoscopy is even lower, due to which the exact numbers are unknown. Suturing the pelvic peritoneum is a common technique in laparoscopic hysterectomy to prevent vaginal cuff infection and organ evisceration in case vaginal cuff dehiscence occurs.^3^, ^4^, ^5^, ^6^ Here, we report an extremely rare case of a strangulated internal hernia caused by an iatrogenic peritoneal band, which was formed by the procedure of suturing the pelvic peritoneum.

## 2 CASE REPORT

**FIGURE 1:**
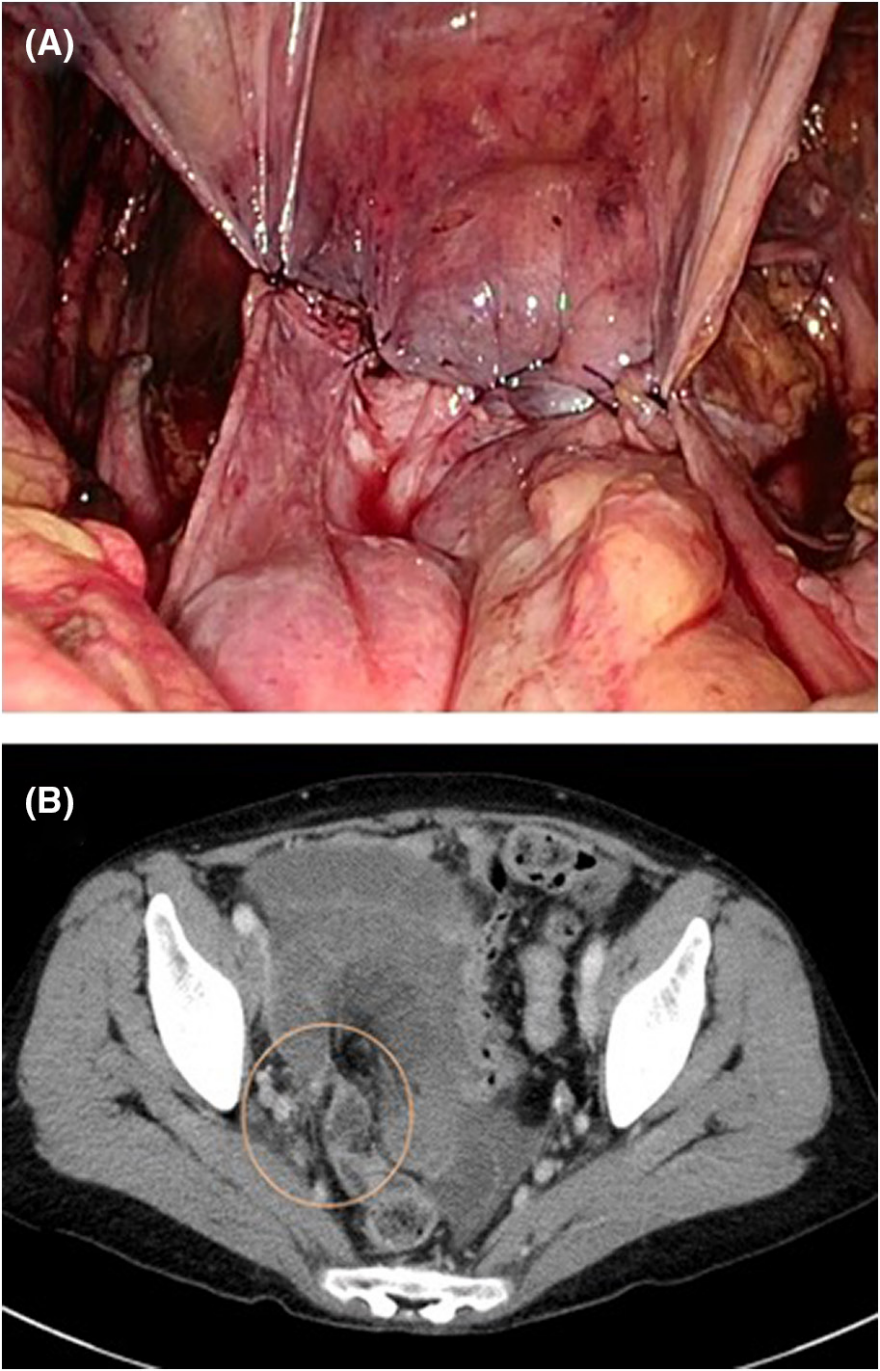
(A) The status of the pelvic peritoneum after the first surgery. (B) Contrast‐enhanced computed tomography scan revealed a closed ileal loop in the right pelvic cavity. The ileum wall showed ischemic change and the mesentery was edematous.

**FIGURE 2:**
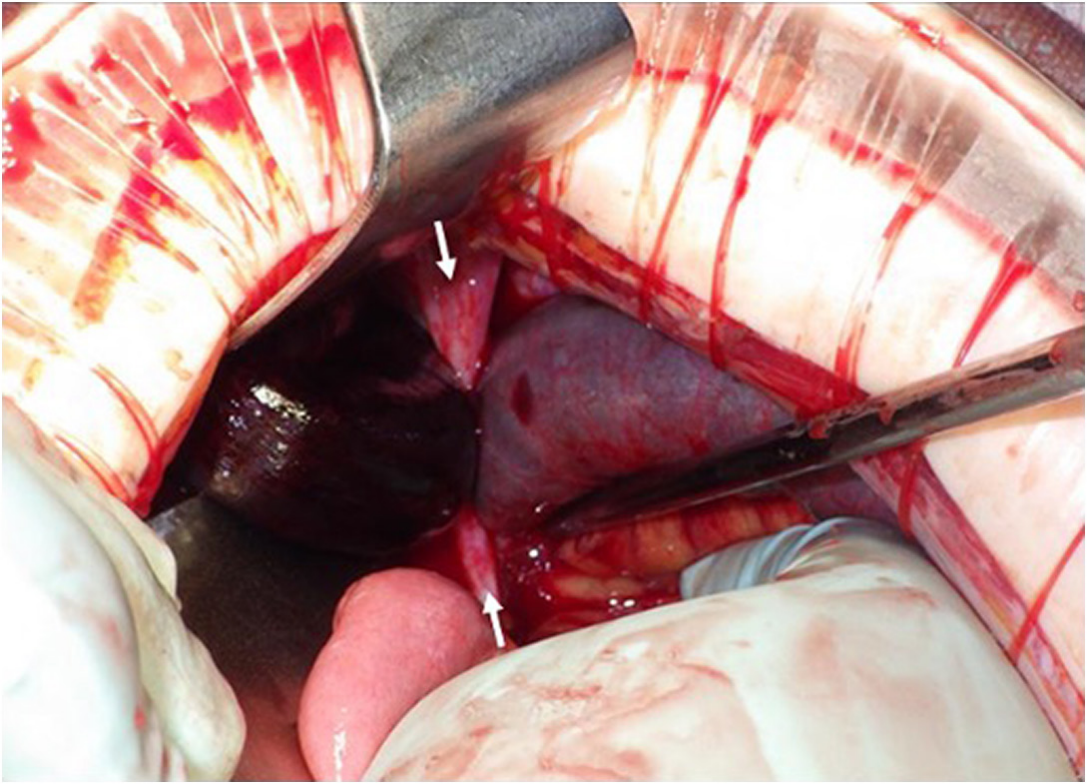
Intraoperative findings. An iatrogenic peritoneum band (white arrows) was forming a closed loop in the right pelvic cavity.

A 64‐year‐old woman, gravida 3, para 3, visited our hospital for treatment of a 17‐cm large uterine fibroid in the cervix. The patient was on steroid therapy for neuromyelitis optica. She had no history of any abdominal surgery. The patient underwent total laparoscopic hysterectomy and bilateral salpingo‐oophorectomy. Due to the uterine size and the fibroid location, the surgery required 6 hours, which was over twice as long as our average operation time. Furthermore, the dissection between the bladder and the cervix was very difficult because of the disturbance in the laparoscopic view, which was severely restricted by the uterine size. As a result, a bladder injury (a 1.5 cm hole) occurred, which was sutured. Subsequently, the hysterectomy proceeded in order. We sutured the pelvic peritoneum with a 2‐0 absorbable monofilament using running suture to cover the raw edge of the closed vagina following the closure of the vaginal cuff. We then applied a spray‐type antiadhesion material to the defects in the pelvic peritoneum (Figure 1A). Postoperative recovery was uneventful, and the patient had no abdominal pain or signs of infection. She was discharged on postoperative day 8 after a bladder leak test through cystography. On postoperative day 23, she visited our hospital for the first time after being discharged. Transvaginal ultrasound did not show ascites or hematoma. However, on postoperative day 29, she returned to our hospital due to sudden‐onset abdominal pain and nausea. On examination, she had rebound tenderness. The blood gas analysis showed lactic acidosis and compensatory alkalemia; the blood pH was 7.55; and lactate was 3.2 mmol/L. Contrast‐enhanced computed tomography revealed a closed loop in the small intestine and was poorly enhanced (Figure 1B). Based on these findings, the patient was diagnosed with strangulated bowel obstruction and emergency surgery was performed. Intraoperatively, we identified that a retroperitoneum band in the right pelvic cavity was the cause of the strangulated ileus (Figure 2). The knot of monofilament found in the peritoneum band suggested that it was a part of the pelvic peritoneum that had been sutured during the previous surgery. Ischemia of the small intestine was severe and required resection of a 1‐meter‐long portion.

Because the patient was on steroid therapy, her food intake was delayed until the 7th postoperative day. Otherwise, the postoperative course was uneventful, and the patient was discharged 10 days after the surgery. The patient was followed up until 3 months after the second surgery without recurrence of intestinal obstruction.

## 3 DISCUSSION

We experienced a rare case of total laparoscopic hysterectomy with severe postoperative complication which needed to be resected small bowel due to the ischemic change. The procedure of peritoneum closure to prevent unfavorable postoperative complication conversely led to another worse consequence. To our knowledge, this is the first case report in which an internal hernia with strangulated ileus was caused by an iatrogenic peritoneal band after laparoscopic hysterectomy.

In gynecologic laparoscopic surgery, intestinal obstruction can be caused by the following in general: port site hernia, the use of barbed sutures, the use of a gelatin‐thrombin matrix sealant, and formation of postoperative adhesions.^6^, ^7^, ^8^ However, internal hernia with strangulated ileus caused by an iatrogenic retroperitoneal band is exceedingly rare. As the technique of suturing retroperitoneum for preventing vaginal cuff related post‐surgical complication is common in laparoscopic hysterectomy, the surgeon should keep in mind that it is possible to induce strangulated ileus.

Internal hernias account for 5.8% of the causes of small intestine obstruction, with a high overall mortality.^9^ Internal hernias are subcategorized as either acquired or congenital defects. Congenital hernia orifices include normal foramina or recesses and abnormal apertures arising from anomalies of internal rotation or peritoneal attachment.^10^ In contrast, acquired hernia orifices are caused by inflammation, trauma, or previous surgery such as gastric bypass for bariatric treatment and liver transplantation. As for gynecologic surgery, strangulated small bowel obstruction cases caused by isolated obturator nerve and pelvic vessels after pelvic lymphadenectomy were reported.^11^ These reported cases seem to be a similar mechanism to our case.

In the present case, we identified that the peritoneal band causing the strangulated ileus was a part of the pelvic peritoneum sutured in the previous surgery from the remaining knot in the band. It is assumed that, because we sutured the pelvic peritoneum despite the large defect due to the cervical leiomyoma, the sutured site might have been exposed to a stronger tension than normal. As a result, the sutured peritoneum ruptured and formed a peritoneum band. Iatrogenic structure that was constructed by the vulnerable peritoneum and sutured monofilament were thought to be constructed the orifice in our case.

Although suturing of the pelvic peritoneum is not a mandatory procedure in laparoscopic hysterectomy, previous case reports and case series have described its utility. It is reported that suturing the pelvic peritoneum can reduce the risk of hematoma, infection, and organ evisceration in case of vaginal cuff dehiscence.^3^, ^4^, ^5^ However, in cases where suturing the peritoneum requires strong tension, such as in large peritoneal defects, this procedure should be reconsidered. Besides, suturing only the middle part of the pelvic peritoneum to cover the closed vaginal cuff and leaving the side walls open may cause an internal hernia. Therefore, in such case with large peritoneum defect, sheet‐type adhesion barrier can be useful to cover the defects in the pelvic peritoneum and prevent intestinal evisceration.

## 4 CONCLUSIONS

Suturing the pelvic peritoneum is one of the common techniques in laparoscopic hysterectomy. However, it may lead to the formation of an iatrogenic peritoneum band, which introduces the risk of internal hernia with strangulated ileus.

## AUTHOR CONTRIBUTIONS

Concept and design: Sakurai S, Suzuki Y. Drafting of the manuscript: Sakurai S, Suzuki Y. Critical revision of the manuscript: Sakurai S, Suzuki Y, Nagai K, Ishidera Y, Nakagawa K, Miyagi E. In charge of patient: Sakurai S, Suzuki Y, Nagai K, Nakagawa K. Supervision: Suzuki Y.

## FUNDING INFORMATION

None.

## CONFLICT OF INTEREST

The authors declare that they have no conflicts of interest.

## ETHICAL APPROVAL

Ethical approval for this study was provided by the Institutional Research Ethics Committee of Yokohama City University School of Medicine. The approval No. is A200600011.

## CONSENT

The authors certify that they have obtained all appropriate patient consent forms. In the form the patient has given her consent for her images and other clinical information to be reported in the journal. The patients understand that their names and initials will not be published and due efforts will be made to conceal their identity, but anonymity cannot be guaranteed.

## Data Availability

All data included in this case report are based on the clinical record. We will make available data (images and reports) upon request.
